# Regulation of Mcl-1 by SRSF1 and SRSF5 in Cancer Cells

**DOI:** 10.1371/journal.pone.0051497

**Published:** 2012-12-17

**Authors:** Hannah L. Gautrey, Alison J. Tyson-Capper

**Affiliations:** Institute of Cellular Medicine, Faculty of Medical Sciences, Newcastle University, Framlington Place, Newcastle upon Tyne, United Kingdom; University of Nebraska Medical Center, United States of America

## Abstract

Up-regulation of the apoptosis-regulatory gene *Mcl-1* (myeloid cell leukemia-1) occurs in different cancer types and is linked with drug resistance to cancer therapies. It is well known that Mcl-1 pre-mRNA undergoes alternative splicing events to produce two functionally distinct proteins, Mcl-1_S_ (pro-apoptotic) and Mcl-l_L_ (anti-apoptotic); the latter isoform is predominant in different cancers including breast and ovarian cancer cells. In the present study we report that the RNA-binding protein (RBP) and proto-oncogene SRSF1 (serine and arginine-rich splicing factor 1) influences splicing of Mcl-1 in both MCF-7 and MDA-MB-231 breast cancer cells and JAR choriocarcinoma cells; we also show for the first time that another RBP SRSF5 affects splicing of Mcl-1 in the MCF-7 cells. Moreover, we report that SRSF1 is involved in other aspects of Mcl-1 regulation with knockdown of SRSF1, by RNAi, resulting in a significant decrease in Mcl-1 protein levels in MCF-7 cells but an increase in JAR cells, respectively, by potentially affecting protein stability and translation of Mcl-l. The key findings from this study highlight the importance of the cellular context of different cancer cells for the function of multifunctional RBPs like SRSF1 and have implications for therapeutic approaches employed to target Mcl-1.

## Introduction

**Figure 1:**
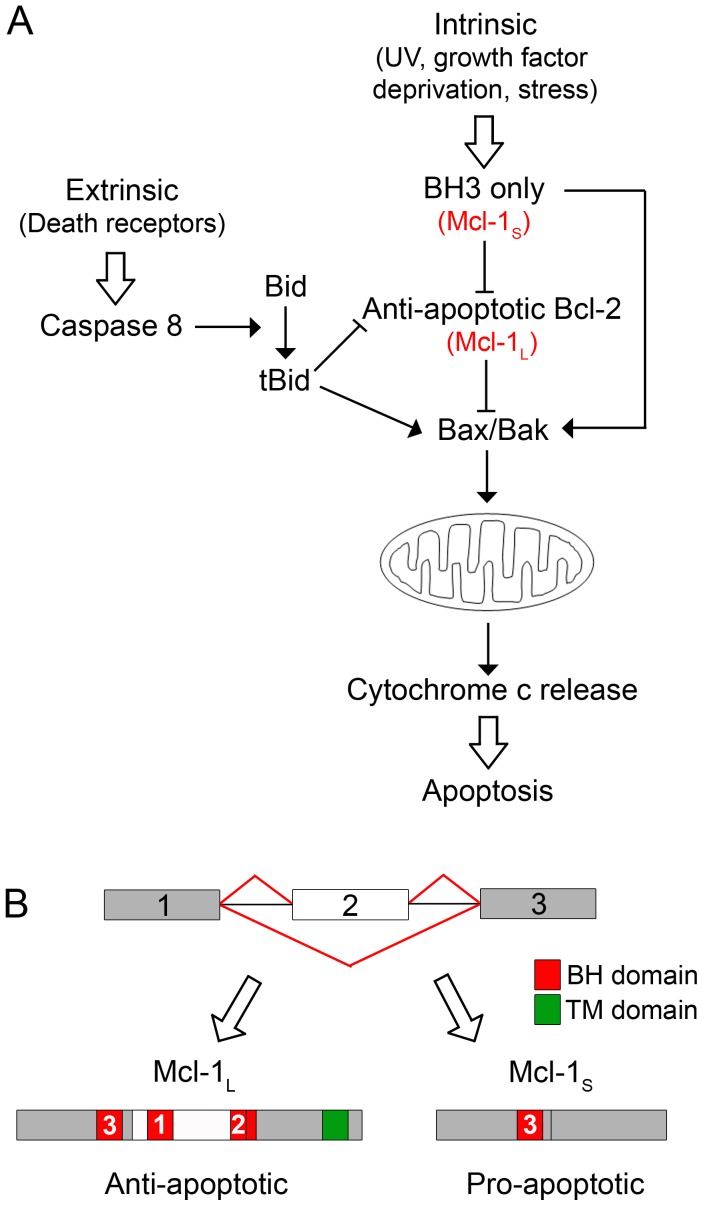
Pathway to show how Mcl-1, a member of the Bcl-2 family, influences apoptosis. (**A**) The intrinsic (mitochondrial) cell death pathway is controlled by the Bcl-2 protein family, including BH3-only proteins, anti-apoptotic Bcl-2 proteins, Bax, Bak and Bid. (**B**) The *Mcl-1* gene consists of three exons; the anti-apoptotic Mcl-1_L_ and the pro-apoptotic Mcl-1_S_ protein isoforms result from inclusion and skipping of exon 2 (open box), respectively.

Apoptosis or programmed cell death is an important process involved in normal development and tissue homeostasis, and its deregulation can result in cancer. A significant number of apoptosis factors have been shown to be regulated by alternative splicing; this includes the Bcl-2 protein family which controls the intrinsic (mitochondrial) cell death pathway [1], [2], Figure 1A. The Bcl-2 family contains both pro-apoptotic and anti-apoptotic proteins, and it is the balance between the two which determines whether the pathway is activated [3], [4]. The Bcl-2 family can be subdivided into three groups based on their structure and function. The anti-apoptotic Bcl-2 proteins contain multiple Bcl-2 homology (BH) domains and so are structurally similar to Bcl-2, which is also a member of this group. The pro-apoptotic Bcl-2 proteins are split into two subgroups, the first group are also structurally similar to Bcl-2 with multiple BH domains, and include the proteins Bak and Bax. The second group of pro-apoptotic proteins only contain the BH3 domain. Apoptosis is triggered when the pro-apoptotic proteins Bak and Bax cause mitochondrial outer membrane permeabilisation. The anti-apoptotic Bcl-2 family members prevent this by binding to the pro-apoptotic proteins Bax and Bak. The BH3-only proteins can activate apoptosis through two routes firstly through direct activation of Bak and Bax, and secondly by binding to the anti-apoptotic proteins, allowing the release of Bak and Bax.

Mcl-1 is a member of the Bcl-2 family of apoptosis regulators. Overexpression of Mcl-1 has been found in a wide range of cancer tissues [5], [6], [7], as well as cancer cell lines [8]. In addition, increased expression of Mcl-1 has been associated with poor prognosis in breast cancer [9]. Mcl-1 also appears to be an important factor involved in resistance to cancer therapies, and its downregulation has proved effective at inducing apoptosis [7], [10], [11], [12].

The *Mcl-1* gene contains three exons and encodes two proteins, the anti-apoptotic Mcl-1_L_ and the pro-apoptotic Mcl-1_S_ [13], [14]. The full length transcript containing all three exons encodes Mcl-1_L,_ which contains BH1, 2, and 3 as well as a TM domain. This results in an anti-apoptotic Bcl-2 protein being produced. Mcl-1_S_ has the second exon spliced out which results in a downstream shift in the reading frame leaving only the BH3 domain remaining (Figure 1B). Mcl-1_S_ appears to exert its pro-apoptotic effect in a similar way to other BH3-only proteins by binding to anti-apoptotic Bcl-2 proteins, and more specifically Mcl-1_S_ binds only to Mcl-1_L_ [13], [15].

A switch in the alternative splicing of Mcl-1 has so far been shown to occur in breast and ovarian cancer, with there being an increase in the anti-apoptotic Mcl-1_L_ isoform in cancer tissues [16]. Despite this, very little is known about the mechanism that regulates the switch in splicing or the splicing factor proteins involved in the inclusion or exclusion of the second exon. So far only two members of the SR protein family, SRSF1 and 3, have been identified as affecting alternate splicing of Mcl-1 [17]. With relevance to this study a range of different splicing factors have been shown to have altered expression in cancer tissues [18]; these include SRSF1 [19] and SRSF3 [20], which are upregulated in a wide range of cancers and have been identified as proto-oncogenes, and SRSF5 which is overexpressed in breast cancer [21].

The aim of the present work was to investigate how Mcl-1 is regulated in cancer cells and identify cell specific RNA binding proteins (RBPs) involved in promoting the inclusion of the second exon of the *Mcl-1* gene. This was achieved by using gene specific knockdown of a range of different RBPs followed by the measurement of the levels of the splice-specific isoforms.

## Materials and Methods

### Cell Culture

**Figure 2:**
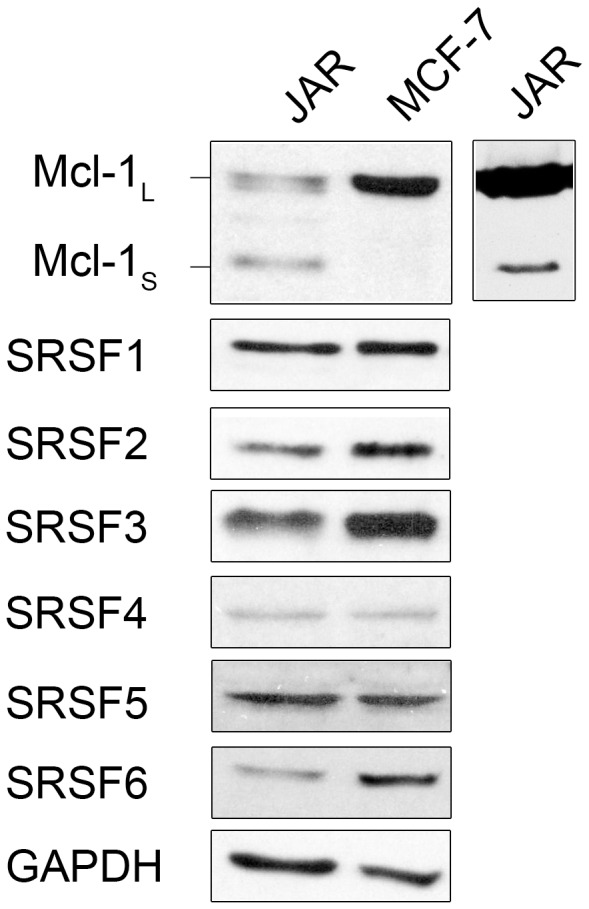
Expression of Mcl-1 and SR proteins in MCF-7 and JAR cells. Top panel shows Mcl splice isoforms in commercially available JAR cell lysate, MCF-7 cells and JAR cells. Detection by western blotting of Mcl-1 splice variants, SRSF1-6 and the loading control GAPDH in 40 ug of total cell lysate from MCF-7 and JAR cells.

Two different cancer cell lines were initially selected for this study (Figure 2), breast cancer adenocarcinoma MCF-7 cells (described as having a low invasion phenotype *in-vitro*) and choriocarcinoma JAR cells; another breast cancer cell MDA-MB-231 cells (described as having an invasive phenotype *in-vitro*) was added to the study for comparison (ATCC, LCG). MCF-7 and MDA-MB-231 cells were maintained in DMEM (Sigma-Aldrich) containing 10% foetal calf serum, L-glutamine and penicillin streptomycin. JAR cells were maintained in RPMI-1640 (ATCC, LCG) containing 10% foetal calf serum and penicillin streptomycin. Cells were incubated at 37°C with 5% CO_2._ Cells were treated with of 20 nM of rapamycin, 35 µg/ml of cycloheximide or 1 µg/ml protease inhibitor (Sigma-Aldrich) where indicated.

### Gene Knockdown by RNAi

All cells were reverse transfected with Silencer® Select siRNA (Ambion; Table 1), *Silencer*® Negative Control siRNA (Ambion), *Silencer*® Select GAPDH Positive Control siRNA (Ambion) and siPORT™ *NeoFX*™ Transfection Agent (Ambion) using the following protocol optimised according to the manufacturers instructions. 5.9×10^4^ MCF-7 cells and 4×10^4^ JAR cells were plated into each well of a 24 well plate. JAR cells were transfected with 4 ul/ml and MCF-7 and MDA-MB-231 cells with 2 ul/ml of siPORT™ *NeoFX*™ transfection agent; all cells were transfected with 6 nM siRNA. Cells and siRNA were incubated together at 37°C in an atmosphere of 5% CO_2_. RNA was collected 48 and 72 hours post transfection and protein collected 72 hours post transfection. In all experiments levels of knockdown by RNAi were assessed at the RNA and protein level by PCR and immunoblotting, as described.

**Table 1:** siRNA sequences.

Targetgene	Sense	Antisense
SRSF1 (1)	GGAUAACACUAAGUUUAGAtt	UCUAAACUUAGUGUUAUCCag
SRSF1 (2)	GCAUCUACGUGGGUAACUUtt	AAGUUACCCACGUAGAUGCgg
SRSF2 (1)	GCACUAUCCUCUUAGAGAAtt	UUCUCUAAGAGGAUAGUGCat
SRSF2 (2)	GCACGAAGGUCCAAGUCCAtt	UGGACUUGGACCUUCGUGCgg
SRSF3	GCAACAAGACGGAAUUGGAtt	UCCAAUUCCGUCUUGUUGCca
SRSF4	GCAUAAAAGUAAGAGCAAAtt	UUUGCUCUUACUUUUAUGCtt
SRSF5 (1)	CCACCUGUAAGAACAGAAAtt	UUUCUGUUCUUACAGGUGGag
SRSF5 (2)	GGACGAUACUCUGACCGUUtt	AACGGUCAGAGUAUCGUCCtc
SRSF6 (1)	CAAAUGAGGGUGUAAUUGAtt	UCAAUUACACCCUCAUUUGtt
SRSF6 (2)	CGUUCUCGAUCAAAAGGCAtt	UGCCUUUUGAUCGAGAACGtg
Tra2B	GGAGGAUACAGAUCACGUUtt	AACGUGAUCUGUAUCCUCCac
SREK1 (1)	CCAACAAUGCAAUAGUAAAtt	UUUACUAUUGCAUUGUUGGag
SREK1 (2)	GGCCCUUGCUUUUAAUGGAtt	UCCAUUAAAAGCAAGGGCCct

Nomenclature and nucleotide sequences of the siRNA oligonucleotides used in this study.

### Western Immunoblotting

Cells were washed in ice cold PBS and then collected in RIPA buffer (25 mM Tris-HCl,pH 7.6, 150 mM NaCl, 1% NP-40, 0.1% sodium deoxycholate and 0.1% SDS) for protein quantification followed by the addition of sample buffer and then heat denatured at 95°C for 5 minutes. Protein lysates were separated using 12% SDS-polyacrylamide gel electrophoresis (PAGE) gels and transferred electrophoretically onto a nitrocellulose membranes. Membranes were blocked for 1 hour with PBS containing 10% dried milk powder, and probed with either mouse anti-Mcl-1 (1∶500, Santa Cruz), rabbit anti-GAPDH (1∶1000, Santa Cruz), mouse anti-SF2/ASF (1∶1000, SRSF1, Zymed), rabbit anti-SC35 (1∶100, SRSF2, Abgent), mouse anti-SRp20 (1∶1000, SRSF3, Zymed), rabbit anti-SRp75 (1∶500, SRSF4, Abcam), goat anti-SRp40 (1∶500, SRSF5, Zymed), rabbit anti-SRp55 (1∶2000, SRSF6, Aviva Systems Biology) overnight at 4°C. After washing in PBS the appropriate HRP-conjugated secondary antibodies diluted in PBS were added. ECL or Super Signal Femto ECL (Pierce) were used for protein detection. Where indicated, densitometric analysis was performed using a UVP gel documentation system and quantification performed using ImageJ software. Data was subsequently analysed using a one-way ANOVA with Tukeys’ multiple comparison test.

### RNA Isolation and Semi-quantitative RT-PCR

**Figure 3:**
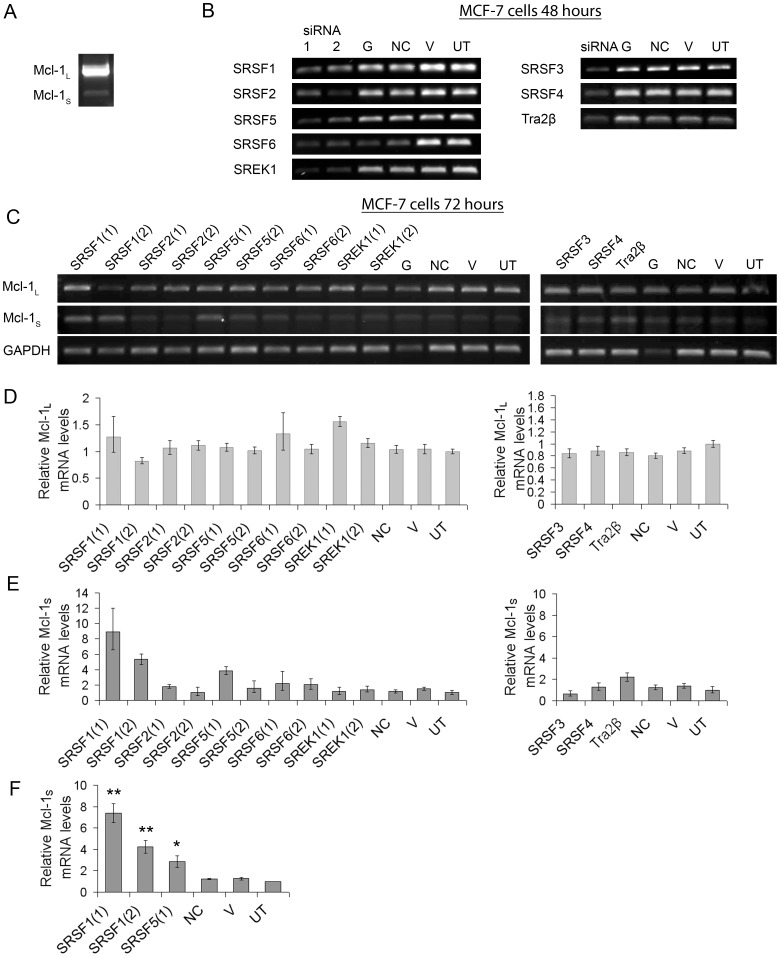
Knockdown of RNA binding proteins in MCF-7 cells and their effect on the mRNA levels of Mcl-1 splice isoforms. MCF-7 cells were transfected with SRSF1-6, Tra2β, SREK1, GAPDH (G) and Negative control (NC) siRNAs, or treated with vehicle (lipid) only (V), or were left untreated (UT). (A) Semi-quantitative RT-PCR showing both Mcl-1 spliced variants. (**B**) Semi-quantitative RT-PCR showing knockdown of RNA binding proteins 48 hours after transfection with siRNAs. (**C**) Semi-quantitative RT-PCR showing levels of the Mcl-1 splice isoforms (Mcl-1_L_ and Mcl-1_S_) and the loading control GAPDH 72 hours after transfection with siRNAs. (**D**) Mcl-1_L_ levels measured by real-time PCR on the same sample shown in (B). (**E**) Mcl-1_S_ levels measured by real-time PCR on the same sample shown in (B). (**F** Mcl-1_S_ levels in sample replicates measured by real-time PCR (mean (n = 3) ± SEM). ** P≤0.01; * P≤0.01.

Total RNA was extracted with RNeasy spin columns (Qiagen) and treated with DNase I (Qiagen) following manufacturers instructions. One µg of total RNA was reverse transcribed using oligo (dT) primers and Superscript II Reverse Transcriptase (RT) (Invitrogen, Life Technologies). PCRs were performed with the cDNA, specific primers (Table 2) and PCR master mix (Promega). Primers for Mcl-1 were designed to be either side of exon 2 so that they could detect both Mcl-1_S_ and Mcl-1_L_ and distinguish the two isoforms by size. (Figure 3A). Negative controls, which lacked RT during the preparation of the cDNA were included to monitor genomic contamination. The cDNA products were separated by electrophoresis on 1.5% (w/v) agarose gels, visualised under UV following ethidium bromide staining.

**Table 2:** Primer sequences.

Target gene	Forward primer	Reverse primer	PCR cycles
Mcl-1	5′-GGACACAAAGCCAATGGGCAGGT-3′	5′-GCAAAAGCCAGCAGCACATTCCTGA-3′	26/36
SRSF1	5′-GCCCCGCAGGGAACAACGAT-3′	5′-CGTCTCGCGGGTCCTCGAAC-3′	26
SRSF2	5′-AGTCTCGGTCCCGCACTCGT-3′	5′-CCAAAGGTGAGTAACCTCCGAGCAG-3′	28
SRSF3	5′-CGTGTGGATTTGAGCCGCCGC-3′	5′-TGGAGATCTGCGACGAGGTGGAGGA-3′	24
SRSF4	5′-GCCAGGTTCCAGACGACGCC-3′	5′-GCGGCTCTTGCCAGCGCTAT-3′	28
SRSF5	5′-TTGCGGATGCACACCGACCT-3′	5′-TCCGGCTCCTGCTCCTGCTT-3′	24
SRSF6	5′-CTTGCGGTCCGCCGTTCGAC-3′	5′-GTATCCACCTCCACCACTGCGG-3′	26
SREK1	5′-TGTGCGGATGGCAGGTGATGAG-3′	5′-TGGGAACGAGATCGACCGCCTT-3′	26
Tra2B	5′-GGAGGCGGTGCGGAGCATTT-3′	5′-TCGCCGCTGTCGCTCATGAC-3′	28
GAPDH	5′-CTGCCGTCTAGAAAAACC-3′	5′-CCAAATTCGTTGTCATACC-3′	20

Nomenclature and nucleotide sequences of the PCR primer used in this study.

### Real-Time PCR

Real-time PCR was performed on cDNA using inventoried TaqMan® assays (Applied Biosystems) and TaqMan® Universal Master Mix II (Applied Biosystems). Taqman assays were selected that were specific for each of the splice variants of Mcl-1 as the probes were designed to span exon boundaries specific to each splice variant (Mcl-1_S_ - exon boundary 1–3, Mcl-1_L_ - exon boundary 1–2), and Taqman GAPDH assay was selected as an endogenous control. The assay was performed in quadruplet and the PCR amplification was performed using the OneStepPlus real-time PCR system (Applied Biosystems). All experiments were performed in triplicate with a minimum of three independent experiments.

### miRNA Isolation and Real-time PCR

Total RNA was extracted with TRIzol®, precipitated with 100% ethanol and then purified on RNeasy spin columns (Qiagen) using only buffer RPE. Total RNA was eluted from the columns with RNase free water. Reverse transcription reaction was performed on 10 ng of total RNA, using TaqMan® MicroRNA Reverse Transcription Kit (Applied Biosystems), and sequence specific RT primers from the TaqMan® Small RNA Assays (Applied Biosystems) according to manufacturers instructions. Separate reverse transcription reactions were performed for each TaqMan® Small RNA Assay on every RNA sample. Real-time PCR was performed on cDNA using inventoried TaqMan® Small RNA Assays and TaqMan® Universal Master Mix II (Applied Biosystems). The assay was performed in triplicate and the PCR amplification was performed using the OneStepPlus real-time PCR system (Applied Biosystems).

### Apoptosis Assay

MCF-7 cells were reverse transfected with siRNA into 96 well plates, and then incubated for 48 hours. MCF-7 cells were then treated with the topoisomerase inhibitor, Etoposide, as previously described [22]. In brief, cells were incubated with serum starvation media (1% FCS) for 18 hours, followed by treatment with 200 µM Etoposide (Sigma Aldrich) for 6 hours [22]. Caspase levels were then measured using Caspase-Glo 3/7 assay (Promega) according to manufacturer’s instructions. Briefly equal volumes of Caspase-Glo 3/7 reagent were added to the wells and incubated at room temperature for 1 hour and then luminescence was measured on a luminometer (BMG).

## Results

### Mcl-1 Splice Isoforms and SR Proteins in Cell Lines

RBPs involved in the control of the alternative splicing event in Mcl-1 were investigated in both JAR and MCF-7 cells. The rationale for using these two cell lines was based on the premise that they have different expression profiles for Mcl-1_L_ and Mcl-1_S_. JAR cells produce both Mcl-1_L_ and Mcl-1_S_, whereas MCF-7 cells only produce high levels of Mcl-1_L_ (Figure 2, top panel). Cell lysates from the same cell lines (Figure 2) were also assessed for expression of a selection of RBPs (SR proteins, SRSF1–6), known to be involved in splice site selection and predicted to have putative RNA-binding sites within exon 2 of the *Mcl-1* gene (http://rulai.cshl.edu/cgi-bin/tools/ESE3/esefinder). Comparisons of the expression levels of these SR proteins in the MCF-7 and JAR cell lines only showed slight increases in SRSF2, 3 and 6 in the MCF-7 cells. Despite there being similar levels of expression, these SR proteins may still be involved in the alternative splicing of Mcl-1, as the activity of SR proteins are controlled by their combined nuclear concentration and activation states in addition to their overall protein levels.

### mRNA Levels of Mcl-1 Splice Isoforms after Knockdown of RNA Binding Proteins

To identify the RBPs involved in the inclusion of the second exon of Mcl-1, siRNA was used to knockdown candidate proteins in MCF-7 cells. The RBPs SRSF1, 2, 5, 6 and SR regulatory protein SREK1 (SFRS12) had two different siRNA which targeted their sequence whereas SRSF3, 4 and Tra2β had one siRNA. An important consideration was to first verify that knockdown of individual SRSFs by RNAi didn’t affect the expression of other family members (Figure S1). Figure 3B demonstrates the knockdown of the RBPs in MCF7 cells 48 hours after transfection, and shows that levels of mRNA for each RBP was reduced by at least one siRNA. An initial screen of the effect of siRNA-mediated knockdown by both semi-quantitative PCR (Figure 3C) and real-time PCR (Figure 3D and 3E) showed that at 72 hours post transfection levels of Mcl-1_S_ increased when cells were depleted of SRSF1(1 and 2) and SRSF5 (1); a slight increase in Mcl-1_S_ was also observed when Tra2β levels were reduced by siRNA. Although the second siRNA that targeted SRSF5 (2) did not produce a similar increase in Mcl-1_S_, the knockdown of SRSF5 was not as effective as with SRSF5 (1) siRNA. Levels of Mcl-1_L_ RNA were also measured (Figure 3C and 3D), but as the expression levels of Mcl-1_L_ mRNA were so high the slight changes produced by the switch in splicing were not observed in the overall mRNA expression. After the initial screen of siRNAs the knockdowns were repeated with SRSF1 (1 and 2) and SRSF5 (1) siRNAs (n = 3), and Figure 2F shows significant up-regulation of Mcl-1_S_ mRNA 72 hours after transfection of these siRNAs into the MCF-7 cell line.

**Figure 4:**
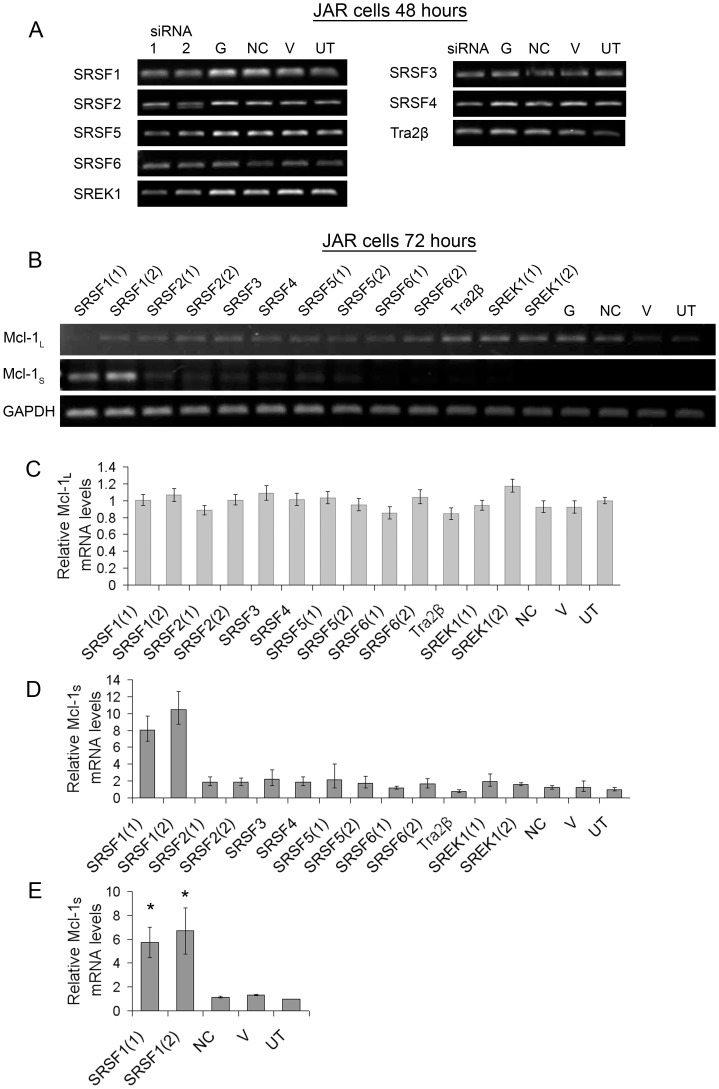
Knockdown of RNA binding proteins in JAR cells and their effect on the mRNA levels of Mcl-1 splice isoforms. JAR cells were transfected with SRSF1-6, Tra2β, SREK1, GAPDH (G) and Negative control (NC) siRNAs, or treated with vehicle (lipid) only (V), or were left untreated (UT). (**A**) Semi-quantitative RT-PCR showing knockdown of RNA binding proteins 48 hours after transfection with siRNAs. (**B**) Semi-quantitative RT-PCR showing levels of the Mcl-1 splice isoforms (Mcl-1_L_ and Mcl-1_S_) and the loading control GAPDH 72 hours after transfection with siRNAs. (**C**) Mcl-1_L_ levels measured by real-time PCR on the same sample shown in (B). (**D**) Mcl-1_S_ levels measured by real-time PCR on the same sample shown in (B). (**E**) Mcl-1_S_ levels in sample replicates measured by real-time PCR (mean (n = 3) ± SEM). * P≤0.01.

To assess which RBPs are involved in this alternative splicing event in JAR cells, the same panel of siRNAs were used to knockdown the RBPs in these cells. Figure 4A shows the knockdown achieved with these siRNAs in JAR cells 48 hours after transfection. The switch in splicing was assessed by measuring the mRNA levels of Mcl-1 by semi-quantitative PCR (Figure 4B) and real-time PCR (Figure 4C and 4D) after 72 hours. Results show a large increase in the level of Mcl-1_S_ after SRSF1 had been knocked down by either SRSF1 (1) or SRSF1 (2). Mcl-1_L_ levels were also measured in the screen, but like the MCF-7 cells no change was observed due to the high levels of Mcl-1_L_ mRNA present. To validate the results of the initial screen the knockdowns with SRSF1 (1 and 2) were repeated (n = 3) and showed a significant increase in the levels of Mcl-1_S_ mRNA after knockdown of SRSF1 (Figure 4E).

### Protein Levels of Mcl-1 Splice Isoforms after Knockdown of RNA Binding Proteins

**Figure 5:**
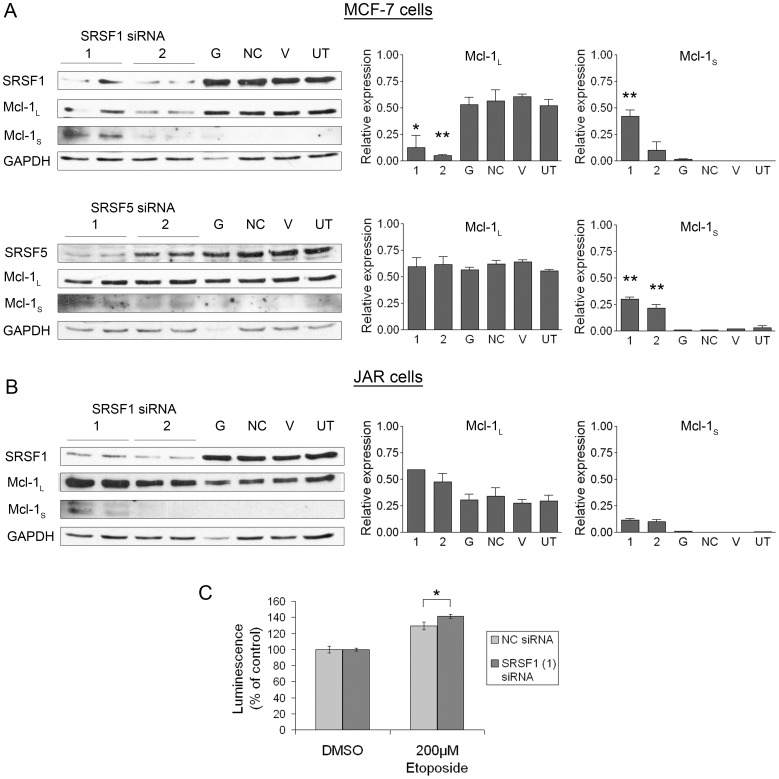
Knockdown of RNA binding proteins and their effect on the protein levels of Mcl-1 splice isoforms. (**A**) MCF-7 cells were transfected with SRSF1 (1 and 2), SRSF5 (1 and 2), GAPDH (G) and Negative control (NC) siRNAs, or treated with vehicle (lipid) only (V), or were untreated (UT). SRSF1, 5, Mcl-1 and GAPDH protein levels were determined by immunoblotting 72 hours after transfection with the siRNAs, using 30µg of total cell lysate. Histograms show densitometric analysis of Mcl-1_L_ and Mcl-1_S_ protein expression. Data are shown as the mean ± SEM. ** indicates P≤0.01; * indicates P<0.05. (**B**) JAR cells were transfected with SRSF1 (1 and 2), GAPDH (G) and Negative control (NC) siRNAs, or treated with vehicle (lipid) only (V), or were untreated (UT). SRSF1, Mcl-1 and GAPDH protein levels were determined by immunoblotting 72 hours after transfection with the siRNAs, using 30 µg of total cell lysate. (**C**) After transfection with SRSF1 (1) and NC siRNA MCF-7 cells were treated with 200 µM etoposide or DMSO control for 6 hours. Induction of apoptosis was assessed by measuring caspase3/7 activity.* indicates P<0.05.

To determine whether the switch in splicing observed in the mRNA was translated into proteins, immunoblotting was used to define the expression pattern of Mcl-1_L_ and Mcl-1_S_ in MCF-7 and JAR cells. It is worthy to note that levels of Mcl-1_S_ and Mcl-1_L_ proteins cannot be visualised, for quantification purposes, at the same exposure time (Figure S4) due to the low levels of endogenous Mcl-1_S_. Knockdown of SRSF1 and SRSF5 in MCF-7 cells is shown in Figure 5A, along with the resulting production of small amounts of Mcl-1_S_, replicating the observations made of Mcl-1_S_ mRNA levels (Figure 3B and 3D). Protein levels of Mcl-1_L_ are also shown after knockdown. The levels of Mcl-1_L_ after treatment with SRSF5 siRNA do not change significantly from the controls, which is consistent with the mRNA levels of Mcl-1_L_ after SRSF5 knockdown (Figure 3B and 3C). Interestingly, the levels of Mcl-1_L_ were significantly reduced after SRSF1 was knocked down compared to the controls in MCF-7 cells. This reduction observed in the protein levels was not observed in Mcl-1_L_ mRNA (Figure 3B and 3C), therefore the reduction in SRSF1 may be affecting the translation or stability of Mcl-1 as well as the alternative splicing event. Initial data from immunoprecipitation experiments suggest SRSF1 may bind to Mcl-1 mRNA in MCF-7 cells (Figure S3 and Methods S1); This observation is in keeping with previous evidence that SRSF1 protein:Mcl-1 RNA complexes exist [17].

Figure 5B shows the effect on protein levels of Mcl-1 after treatment with SRSF1 siRNA in JAR cells. Consistent with the mRNA results, reductions in SRSF1 levels resulted in an increase in the protein level of Mcl-1_S_. In contrast, Mcl-1_L_ protein levels increased after treatment with SRSF1 siRNA, whereas the corresponding mRNA results did not show any change in levels after knockdown (Figure 4B and 4C). Consequently, unlike the MCF-7 cell line, Mcl-1_L_ protein levels increased in response to SRSF1 siRNA rather than reducing, this increase was not statistically significant (Figure 4B). These findings indicate that SRSF1 may be involved in other aspects of Mcl-1 regulation such as translational control or protein stability in JAR cells. Protein levels of Mcl-1_L_ and Mcl-1_S_ were also examined after knockdown with the other RBPs used in the RNA screen in both the MCF-7 and JAR cells, but no changes were observed (data not shown).

We next investigated whether knockdown of SRSF1, which resulted in such a dramatic change in Mcl-1_L_ and Mcl-1_S_ protein ratios in the MCF-7 cells, could affect the induction of apoptosis. We first performed a MTT assay to rule out the possibility that cell proliferation and cell viability may be affected by knockdown of SRSF1 in the MCF-7 cells (Figure S2 and Methods S2). Apoptosis was induced in both the negative control and SRSF1 (1) siRNA treated cells by treatment with etoposide (200 µM), and measured by assessing the resulting caspase 3/7 activity (Figure 5C). The percentage increase in caspase 3/7 activity was slightly higher after knockdown of SRSF1 demonstrating a greater induction of apoptosis.

### Effect of SRSF1 Knockdown on Stability and Translation of Mcl-1_L_

**Figure 6:**
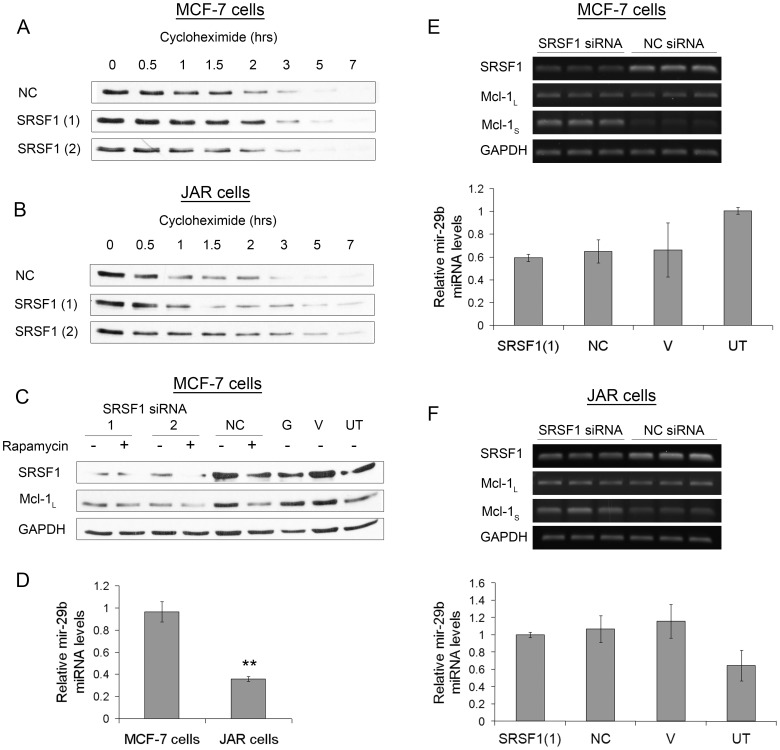
Effect of SRSF1 knockdown on expression, stability and translation of Mcl-1_L_ in MCF-7 and JAR cells. (**A**) MCF-7 and (**B**) JAR cells were transfected with SRSF1 (1 and 2) and negative control (NC) siRNA. Three days after transfection cells were treated with 35 ug/ml cycloheximide and protein samples were collected after 0, 0.5, 1, 1.5, 2, 3, 5 and 7 hours to assess protein stability. Mcl-1_L_ protein levels were then measured by immunoblotting. (**C**) MCF-7 cells were transfected with SRSF1 (1 and 2), GAPDH (G) and negative control (NC) siRNA, or treated with vehicle (lipid) only (V), or were untreated (UT). 48 hours after transfection cells were treated with 20 nM rapamycin for a further 24 hours. Western blotting was then used to assess protein levels of SRSF1, Mcl-1_L_ and GAPDH. (**D**) mir-29b levels measured by real-time PCR in JAR and MCF-7 cell lines (mean (n = 3) ± SEM), ** P≤0.01. Knockdown of SRSF1 in MCF-7 cells (**E**) and JAR cells (**F**) was assessed by semi-quantitative RT-PCR in SRSF1 (1) and negative control (NC) siRNA (upper panels). Levels of mir-29b were measured by real-time PCR in SRSF1 (1) and negative control (NC) siRNA treated MCF-7 cells from (E) and JAR cells from (F) along with GAPDH (G) siRNA, vehicle (lipid) only (V), and untreated (UT) cells (lower panels) (mean (n = 3) ± SEM).

Mcl-1 contains multiple residues within the N-terminal region that can undergo post translational modification, such as ubiquitination and phosphorylation [23]. These modifications can affect the stability of the Mcl-1 protein. Therefore, we went on to assess the stability of Mcl-1 protein after knockdown of SRSF1 in the two cell lines. Cycloheximide treatment was used to block protein translation and Mcl-1_L_ protein levels were assessed over the following 7 hours. Figure 6A shows a very similar rate of loss of Mcl-1_L_ in the MCF-7 cells after treatment with SRSF1 siRNA compared with negative control siRNA. This suggests that there is no change in the stability of Mcl-1_L_ after knockdown of SRSF1 in the MCF-7 cells. In contrast, treatment of the JAR cells with cycloheximide (Figure 6B) resulted in slightly slower rate of loss of Mcl-1_L_ after knockdown of SRSF1, indicating that there may be a slight increase in Mcl-1_L_ protein stability.

As the decrease in the levels of Mcl-1_L_ in the MCF-7 cells after SRSF1 knockdown does not appear to be affecting the stability of the protein, it may be a translational effect. SRSF1 has already been demonstrated to be involved in mTOR translation initiation, where its presence on the RNA results in enhanced recruitment of the mTOR complex to the mRNA [24], [25], [26], [27], [28]. Additionally, Mcl-1 has also been shown to be translationally controlled by mTOR and the mTOR signalling pathway [24], [25], [26], [27], [28]. To further investigate this, MCF-7 cells were treated with the mTOR inhibitor rapamycin. Figure 6C confirms the reduction in Mcl-1_L_ after treatment with rapamycin and shows this reduction in Mcl-1_L_ to be similar to that observed after SRSF1 knockdown. Furthermore, the addition of rapamycin to the SRSF1 knockdown cells did not result in any further decrease in the levels of Mcl-1_L_.

A recent report has also suggested another mechanism by which SRSF1 can control translation, through the processing of miRNAs [29]. One of the miRNAs identified as being upregulated after overexpression of SRSF1 was mir-29b. This miRNA has already been shown to be involved in the translational inhibition of Mcl-1 [30]. In light of this we hypothesised the increase observed in Mcl-1_L_ protein levels in the JAR cells may, in part, be due to decreased levels of mir-29b. As the knockdown of SRSF1 in the MCF-7 cells did not show a similar increase in the levels of Mcl-1_L_ protein but in fact a decrease, we would not expect mir-29b to be involved in the regulation of Mcl-1_L_ in MCF-7 cells. As a result the initial levels of mir-29b might be expected to be higher in JAR cells compared to MCF-7 cells. To investigate this mir-29b was measured in untreated JAR and MCF-7 cells by real-time PCR (Figure 6D). This showed, contrary to expectation, that levels of mir-29b were significantly higher in the MCF-7 cells compared to the JAR cells. To assess whether the knockdown of SRSF1 affected the levels of mir-29b, as over-expression has previously been shown to do this [29], siRNA was used to knockdown SRSF1 in MCF-7 cells (Figure 6E) and JAR cells (Figure 6F). The knockdown was initially performed in MCF-7 cells since they showed the highest initial levels of mir-29b (Figure 6D). Although treatment with the transfection reagent alone seemed to result in a reduction in mir-29b in MCF-7 cells, knockdown of SRSF1 had little effect on the levels of mir-29b in both cell types when compared with the negative control siRNA transfected cells (Figure 6E and 6F).

**Figure 7:**
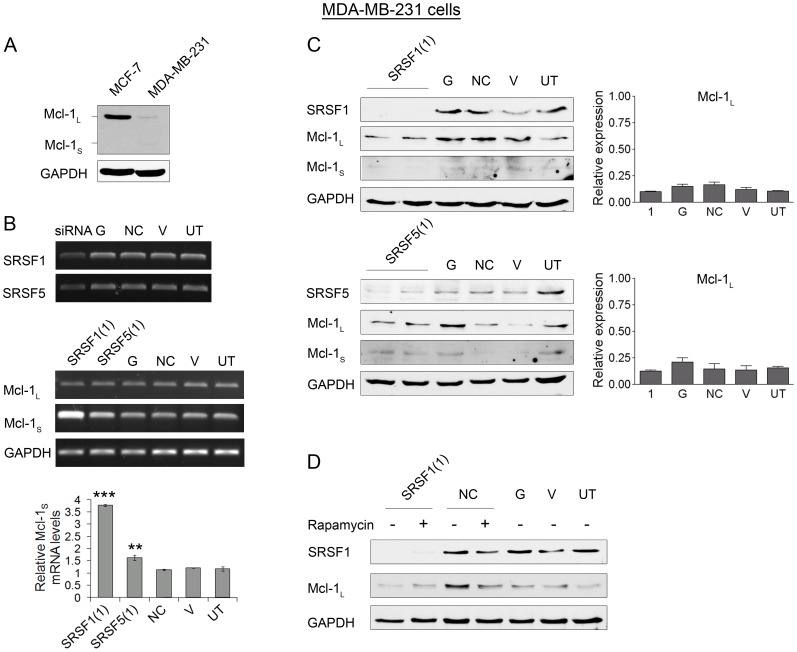
Effect of SRSF1 and SRSF5 knockdown on expression, stability and translation of Mcl-1_L_ in MDA-MB-231 cells. (**A**) Detection of Mcl-1 splice variants and GADPH proteins in MCF-7 and MDA-MB-231 using 40 ug of total cell lysate from both cell types. Histograms show densitometric analysis of Mcl-1_L_ and Mcl-1_S_ protein expression. Data are shown as the mean ± SEM. (**B**) Semi-quantitative RT-PCR showing knockdown of RNA binding proteins 48 hours after transfection with siRNAs. MDA-MB-231 cells were transfected with SRSF1, GAPDH (G) and Negative control (NC) siRNAs, or treated with vehicle (lipid) only (V), or were left untreated (UT). Semi-quantitative RT-PCR showing levels of the Mcl-1 splice isoforms (Mcl-1_L_ and Mcl-1_S_) and the loading control GAPDH 72 hours after transfection with siRNAs. Mcl-1_S_ levels in sample replicates measured by real-time PCR (mean (n = 3) ± SEM). (**C**) MDA-MB-231 cells were transfected with SRSF1, SRSF5, GAPDH (G) and Negative control (NC) siRNAs, or treated with vehicle (lipid) only (V), or were untreated (UT). SRSF1, 5, Mcl-1 and GAPDH protein levels were determined by immunoblotting 72 hours after transfection with the siRNAs, using 30 µg of total cell lysate. (**D**) MDA-MB-231 cells were transfected with SRSF1, GAPDH (G) and negative control (NC) siRNA, or treated with vehicle (lipid) only (V), or were untreated (UT). 48 hours after transfection cells were treated with 20 nM rapamycin for a further 24 hours. Immunoblotting was then used to assess protein levels of SRSF1, Mcl-1_L_ and GAPDH (mean (n = 3) ± SEM). *** indicates P≤0.001; ** P≤0.01.

Regulation of Mcl-1 by SRSF1 and 5 was also investigated in a second breast cancer cell line using MDA-MB-231 cells, described as having an invasive phenotype *in vitro*. Endogenous Mcl-1 protein levels were assessed by western blotting (Figure 7A), indicating that MDA-MB-231 cells express mainly the Mcl-1_L_ protein isoform, but at much lower levels than MCF-7 cells. To determine whether the same RBPs are involved in the splicing of Mcl-1 in MDA-MB-231 cells siRNA against SRSF1 and 5 was used to deplete cells of these two proteins. Figure 7B shows a reduction in the RNA levels of SRSF1 and 5 after 48 hours, and a subsequent increase in the levels of Mcl-1_S_ mRNA after 72 hours. Real time PCR analysis of repeated transfections also demonstrated a significant upregulation of Mcl-1_S_ mRNA 72 hours after transfection with both SRSF1 and 5 siRNAs. Protein levels were also measured by immunoblotting after knockdown of SRSF1 and 5. Figure 7C shows knock down of SRSF1 and 5 proteins 72 hours after transfection. Due to the low levels of Mcl-1_L_ and Mcl-1_S_ in the MDA-MB-231 cell line Super Signal Femto ECL was used to detect the two isoforms, but this failed to show any significant change in the two isoforms after knockdown of SRSF1 or 5, although there was a slight reduction in Mcl-1 protein after knockdown of both SR proteins (Figure 7C). As the mTOR pathway appears to be important in the regulation of Mcl-1 in MCF-7 cells this was also investigated in the MDA-MB-231 cells. Rapamycin was used to inhibit the mTOR pathway in this cell line, but the dramatic reduction that was observed in the MCF-7 cells with rapamycin treatment and SRSF1 knockdown was not less evident with the MDA-MB-231 cells.

## Discussion

The results presented here as well as the study by Moore, M et al [17] provide increasing evidence for the central role of SRSF1 in the control of the alternative splicing of Mcl-1. Previously SRSF1 has been identified as a proto-oncogene with increased expression in a wide variety of tumours [19], and in addition has been demonstrated to be involved in cancer associated changes in alternative splicing. With relevance to breast cancer progression, alternative splicing events mediated by SRSF1 are capable of promoting mammary gland tumorigenesis [31]. More specifically, SRSF1 has been shown to affect the alternative splicing of the tumour suppressor Bin1 [19], [31], kinases (S6K1 and Mnk2) [19], transcripts involved with cell mobility (Ron [31], [32] and Rac1 [33]), proliferation (Cyclin D1 [34]) and apoptosis (Bcl-x [17]) and Bim (also known as BCL2L11) [31]. The involvement of SRSF1 in the production of the anti-apoptotic of Mcl-1 in breast cancer cells adds to the number of cancer specific splicing events controlled by this SR protein.

The other splicing factor identified in this study as influencing the splicing of Mcl-1 was SRSF5. This is the first time this SR protein has been linked with the alternative splicing of Mcl-1. Previously SRSF5 expression has been shown to be increased in breast cancer, where it was associated with changes in alternative splicing of CD44 as well as lymph node metastasis [21]. So its involvement in the production of the anti-apoptotic form of Mcl-1 in the MCF-7 and MDA-MB-231 breast cancer cell lines is consistent with its known upregulation in breast cancer tissue.

Despite the clear involvement of SRSF5 in MCF-7 and MDA-MB-231 breast cancer cells, this SR protein did not appear to be involved in the alternative splicing of Mcl-1 in JAR cells. However, we cannot rule out that this difference is due to SRSF5 not being sufficiently knocked down in the JAR cells, as a result of the reduced transfection efficiency of this cell line. Other SR proteins previously implicated in the splicing of Mcl-1 include SRSF3 and Tra2β. This study found no effect on the splicing pattern of Mcl-1 after knockdown of SRSF3 in either JAR or MCF-7 cells, and only a very slight increase in Mcl-1_S_ mRNA levels in MCF-7 cells after knockdown of Tra2β. In contrast, Moore, et al [17] found that knockdown of SRSF3 and Tra2β in HeLa cells affected the alternative splicing pattern of a Mcl-1 reporter construct. Taken together these findings indicate that different splicing factors are involved in the selection of splice sites in the Mcl-1 transcript in different cellular contexts.

In addition to demonstrating the role of SRSF1 in the alternative splicing of Mcl-1 we also show its involvement in other aspects of Mcl-1 regulation. Knockdown of SRSF1 resulted in a decrease in Mcl-1 in the breast cancer cell lines studied and an increase in the choriocarcinoma cells, and achieved this by both affecting protein stability and translation of Mcl-1. In the JAR cells, knockdown of SRSF1 resulted in a slight increase in the stability of Mcl-1_L_ protein. Mcl-1 is an unstable protein which has a relatively short half life, due to the PEST regions within its N-terminal. This N-terminal region contains residues which are phosphorylated resulting in both increases and decreases in the protein’s stability, as well as sites of ubiquitination and cleavage [23]. All these post-translational modifications of Mcl-1 affect the rate of turnover, and therefore in the future it will be interesting to investigate whether they are targeted by SRSF1, and if so the mechanism involved.

SRSF1 was also shown to be involved in the translational control of Mcl-1 in MCF-7 cells, as the knockdown of SRSF1 had no observable effect on the stability of Mcl-1_L_. The data presented here indicates that this translational effect may be mediated by interactions with the mTOR complex. The mTOR signalling pathway is involved in controlling cell proliferation, growth and survival, and its dysregulation has been shown to commonly occur in cancers. As a result there are a number of mTOR inhibitors currently undergoing clinical trials. Here we confirm previous reports of Mcl-1 being a target of mTOR mediated translation initiation [27], [28], [35], as the mTOR inhibitor rapamycin decreased Mcl-1 protein levels in control cells. There is also growing evidence for the involvement of SRSF1 in activating the mTOR pathway through recruitment of the mTOR complex to the mRNA, resulting in increased translation [24], [25], [26]. In this context, Michlewski *et al* demonstrated that SRSF1 influences translation initiation by enhancing phosphorylation of the translation inhibitor 4E-BP1 and reducing its activity [25]. The results we present here further implicate Mcl-1-_L_ as a potential target for this process of SRSF1 mediated mTOR translation initiation.

Although the knockdown of SRSF1 does appear to affect the stability of Mcl-1_L_ we also investigated a possible mechanism of translational control in this cell line. A previous study had identified mir-29b as being upregulated as a result of enhanced processing, in response to SRSF1 overexpression [29]. This same miRNA had also been demonstrated to control the translation of Mcl-1 [30]. Despite this link we did not find any direct evidence for mir-29b being involved in controlling translation of Mcl-1 in JAR cells, as levels of mir-29b were so much lower in these cells compared to MCF-7 cells. In addition, the knockdown of SRSF1 in MCF-7 cells had very little effect on the levels of mir-29b. Although mir-29b may not be affected by SRSF1 in these cells there remains the possibility that SRSF1 is involved in the processing of another miRNA that also targets Mcl-1 in this cell line, or that SRSF1 is involved in other mechanisms of translational repression.

We also evaluated whether the observed changes in Mcl-1_L_ and Mcl-1_S_ expression as a consequence of SRSF1 knockdown impacted on cell apoptosis; we observed a modest increase in caspase activity in MCF-7 cells. However, despite an increase in Mcl-l_S_ expression when cancer cells were depleted of SRSF1 the relative levels of pro-apoptotic McL-1_S_ compared to those of anti-apoptotic Mcl-1_L_ remained low. This expression profile is consistent with previous work reporting reduced expression of endogenous Mcl-1_S_ in cancer cell lines [36]. It is pertinent that elevated levels of Mcl-l_S_ can induce apoptosis when artificially overexpressed using either a Mcl-1_S_ gene construct [13] or antisense morpholino oligonucleotides that target Mcl-1 pre-mRNA and switch splice-site selection to shift expression from Mcl-1_L_ to Mcl-1_S_ [36]. In light of this it is probable that in the present study the decrease in Mcl-1_L_ explains the slight increase in cell death as opposed to the increase in the pro-apoptotic Mcl-1_S_ isoform. Moreover, it could be reasoned that other pro-apoptotic proteins regulated by SRSF1 may also be involved [31].

In summary, the findings from this study demonstrate the importance of the cellular context for the function of multifunctional RNA binding proteins like SRSF1, and have important implications for therapeutic approaches employed to target Mcl-1. For example, in breast tumour cells with similar cellular environments to the MCF-7 cells, the knockdown of SRSF1 may provide a powerful way of inducing apoptosis, as there is both a switch in splicing and a reduction in translation of Mcl-1_L_ protein. However in a cellular environment similar to the JAR choriocarcinoma cells this dual effect is not observed. Therefore, in the future it is important to try and understand the mechanisms involved in controlling the functions of SRSF1 in different cell types. These may include differences in the phosphorylation state of the RS domain, which controls its subcellular localisation and activation state, the presence of different binding partners and coregulators, or differences in activation of signalling pathways such as the mTOR pathway. Understanding these mechanisms may also provide further opportunities for affecting Mcl-1 splicing, translation and stability, and therefore ways of inducing apoptosis in cancer cells.

## Supporting Information

Figure S1 **Knockdown of RNA-binding proteins.** (**A**) Semi-quantitative RT-PCR showing knockdown 48 hours after transfection with siRNAs in MCF-7 cells and their effect on the mRNA levels of Mcl-1 splice isoforms (Mcl-1_L_ and Mcl-1_S_) and the loading control GAPDH. MCF-7 cells were transfected with SRSF1, 2, 5, 6, SREK1, GAPDH (G) and Negative control (NC) siRNAs, or treated with vehicle (lipid) only (V), or were left untreated (UT). (**B**) Semi-quantitative RT-PCR of the loading control GAPDH 48 hours after JAR cells were transfected with SRSF1-6, Tra2β, SREK1, GAPDH (G) and Negative control (NC) siRNAs, or treated with vehicle (lipid) only (V), or were left untreated (UT).
(TIF)

Figure S2 **Cell proliferation after SRSF1 knockdown.** Cell proliferation was determined by MTT assay 72 hours after MCF-7 cells were transfected with SRSF1 (1) and Negative control (NC) siRNAs. Results show mean (n = 6) ± SEM.
(TIF)

Figure S3 **SRSF1 interactions with Mcl-l mRNA.** Immunoprecipitation with anti-SRSF1 antibody (SRSF1 Ab), an isotype control antibody (IgG Ab) or without antibody (No Ab) was performed with MCF-7 cell lysate. Bound Mcl-1 mRNA transcripts were detected by RT-PCR, MCF-7 total RNA was also used as a positive control.
(TIF)

Figure S4 **Expression of Mcl-1 proteins in MCF-7 cells.** (**A**) Detection by immunoblotting of Mcl-1_L_ protein and the loading control GAPDH in 40 ug of total cell lysate from MCF-7 using ECL detection reagent (Pierce). (**B**) Detection of Mcl-L_S_ on the same membrane was achieved using an alternative and more sensitive procedure using SuperSignal West Femto ECL detection reagent (Pierce).
(TIF)

Methods S1 **Immunoprecipitation and RNA Isolation.** MCF-7 cells were washed in ice cold PBS and then collected in lysis buffer (10 mM Tris-HCl (pH 7.5), 150 mM NaCl, 0.5% NP-40, 1% Triton X-100) containing protease inhibitors (Sigma-Aldrich). Dynabeads Protein A (Invitrogen) were incubated for 3 hours at 4°C with 10 µg of antibody (mouse anti-SF2/ASF (SRSF1, Zymed) and mouse IgG control (Santa Cruz)) diluted in lysis buffer or with lysis buffer alone. Beads were then washed three times with lysis buffer before being incubated for 1 hour at 4°C with the cell lysate. Beads were then washed five times with lysis buffer before the immunoprecipitated RNA was collected in Trizol reagent (Invitrogen) according to manufacturer’s instructions. The RT-PCR was performed as before but half of the RNA obtained from the immunoprecipitation was used in each reaction.
(DOCX)

Methods S2 **MTT Cell Proliferation Assay.** MCF-7 cells were seeded and transfected with siRNA as before. After 72 hours proliferation was measured using TACS MTT Cell Proliferation Assay (Trevigen), according to manufacturer’s instructions.
(DOCX)
