# Supplementary figures and images for: Regulation of Mcl-1 by SRSF1 and SRSF5 in Cancer Cells
Source: PLoS One. 2012 Dec 17;7(12):e51497. doi: 10.1371/journal.pone.0051497 (PMC3524227; doi:10.1371/journal.pone.0051497)

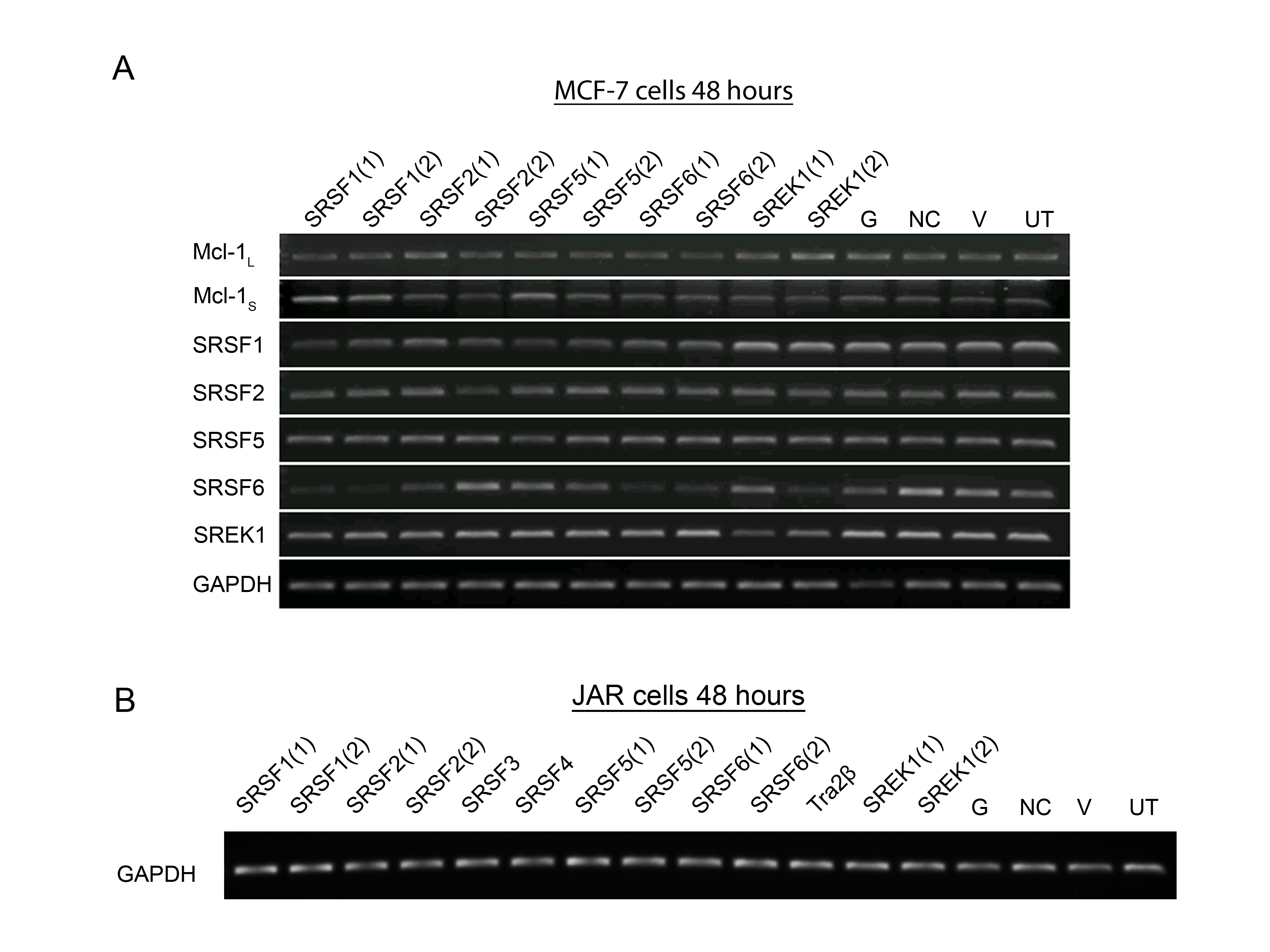

Supplement: Figure S1 — Knockdown of RNA-binding proteins. (A) Semi-quantitative RT-PCR showing knockdown 48 hours after transfection with siRNAs in MCF-7 cells and their effect on the mRNA levels of Mcl-1 splice isoforms (Mcl-1L and Mcl-1S) and the loading control GAPDH. MCF-7 cells were transfected with SRSF1, 2, 5, 6, SREK1, GAPDH (G) and Negative control (NC) siRNAs, or treated with vehicle (lipid) only (V), or were left untreated (UT). (B) Semi-quantitative RT-PCR of the loading control GAPDH 48 hours after JAR cells were transfected with SRSF1-6, Tra2β, SREK1, GAPDH (G) and Negative control (NC) siRNAs, or treated with vehicle (lipid) only (V), or were left untreated (UT). (TIF) [file pone.0051497.s001.tif]

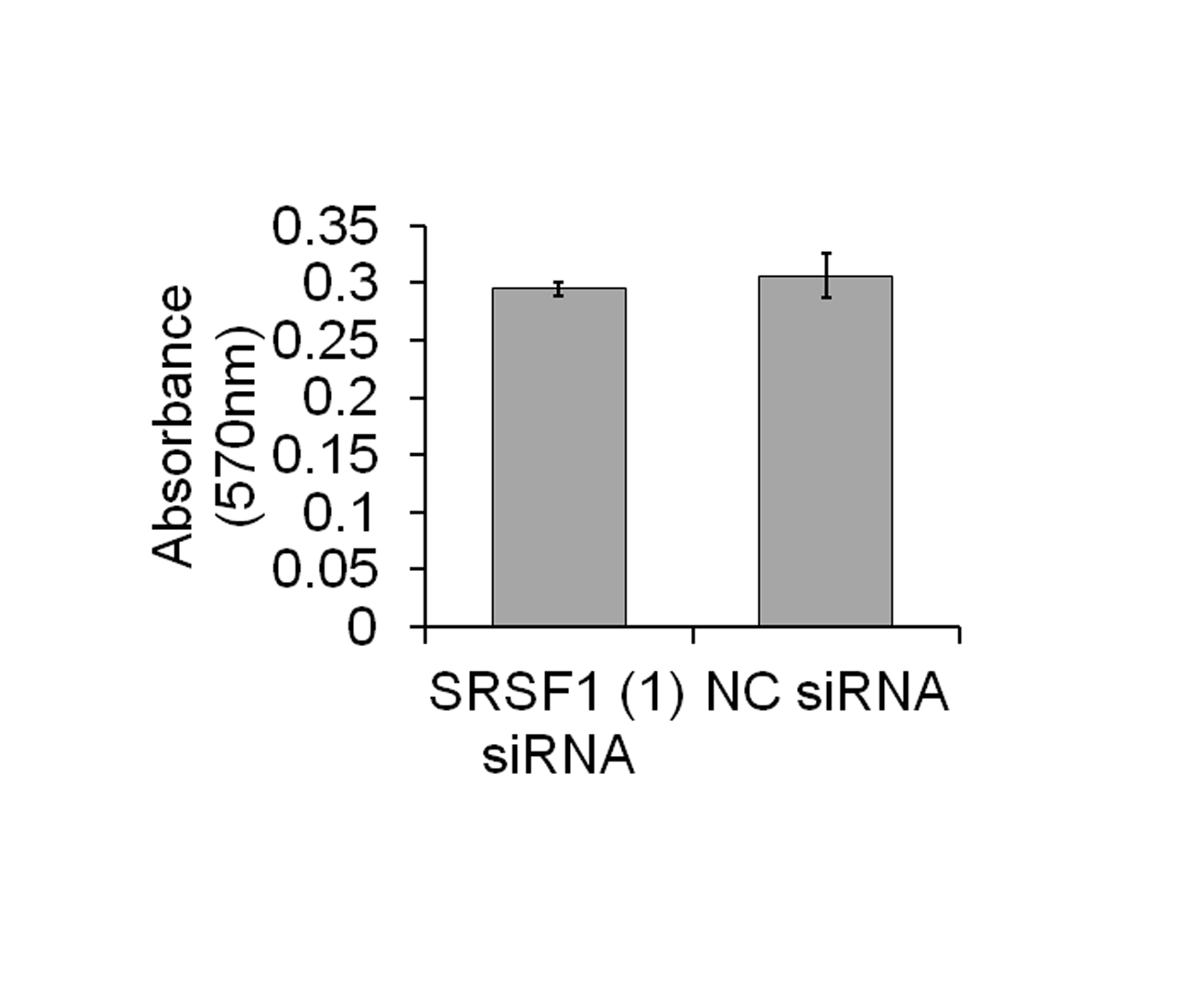

Supplement: Figure S2 — Cell proliferation after SRSF1 knockdown. Cell proliferation was determined by MTT assay 72 hours after MCF-7 cells were transfected with SRSF1 (1) and Negative control (NC) siRNAs. Results show mean (n = 6) ± SEM. (TIF) [file pone.0051497.s002.tif]

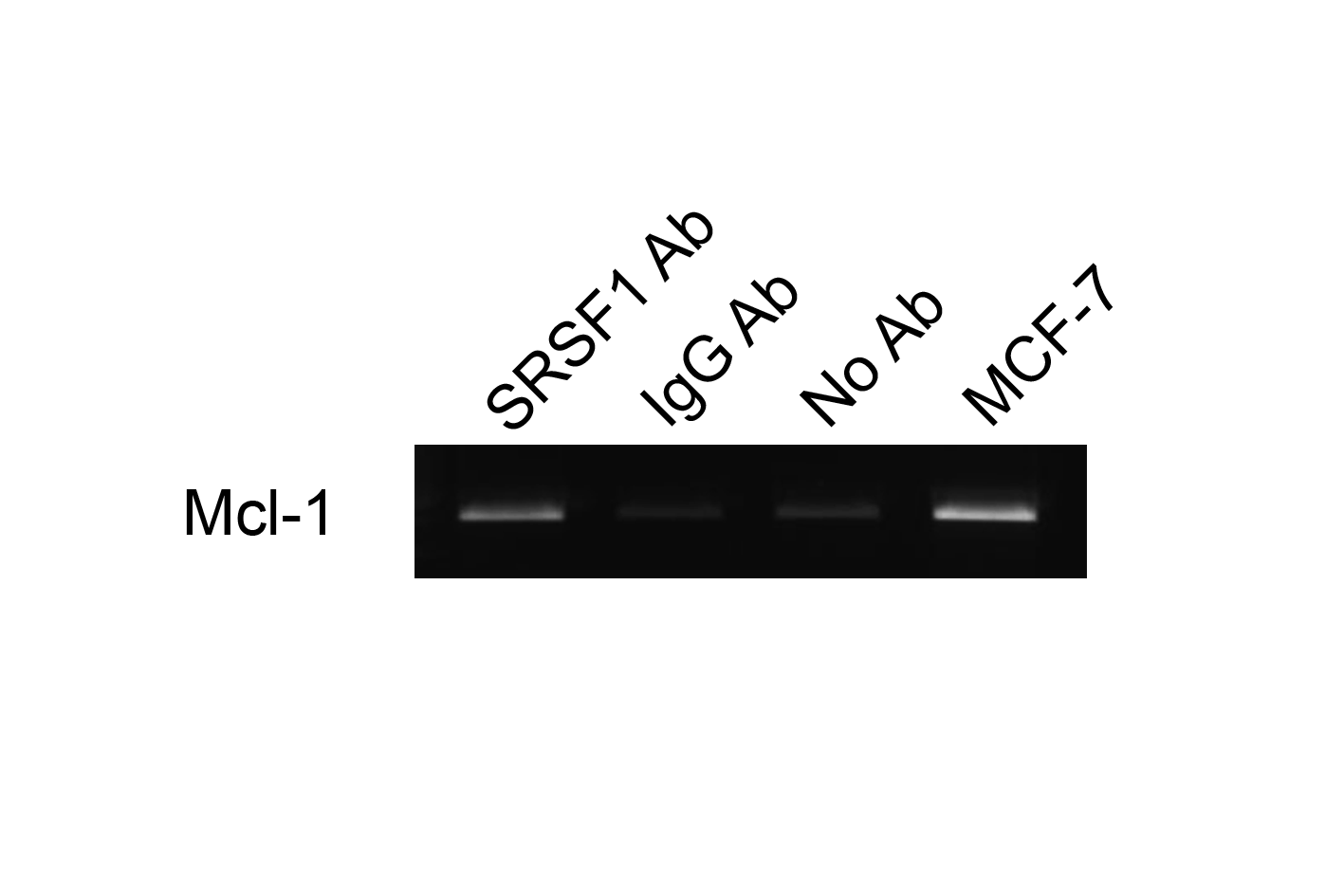

Supplement: Figure S3 — SRSF1 interactions with Mcl-l mRNA. Immunoprecipitation with anti-SRSF1 antibody (SRSF1 Ab), an isotype control antibody (IgG Ab) or without antibody (No Ab) was performed with MCF-7 cell lysate. Bound Mcl-1 mRNA transcripts were detected by RT-PCR, MCF-7 total RNA was also used as a positive control. (TIF) [file pone.0051497.s003.tif]

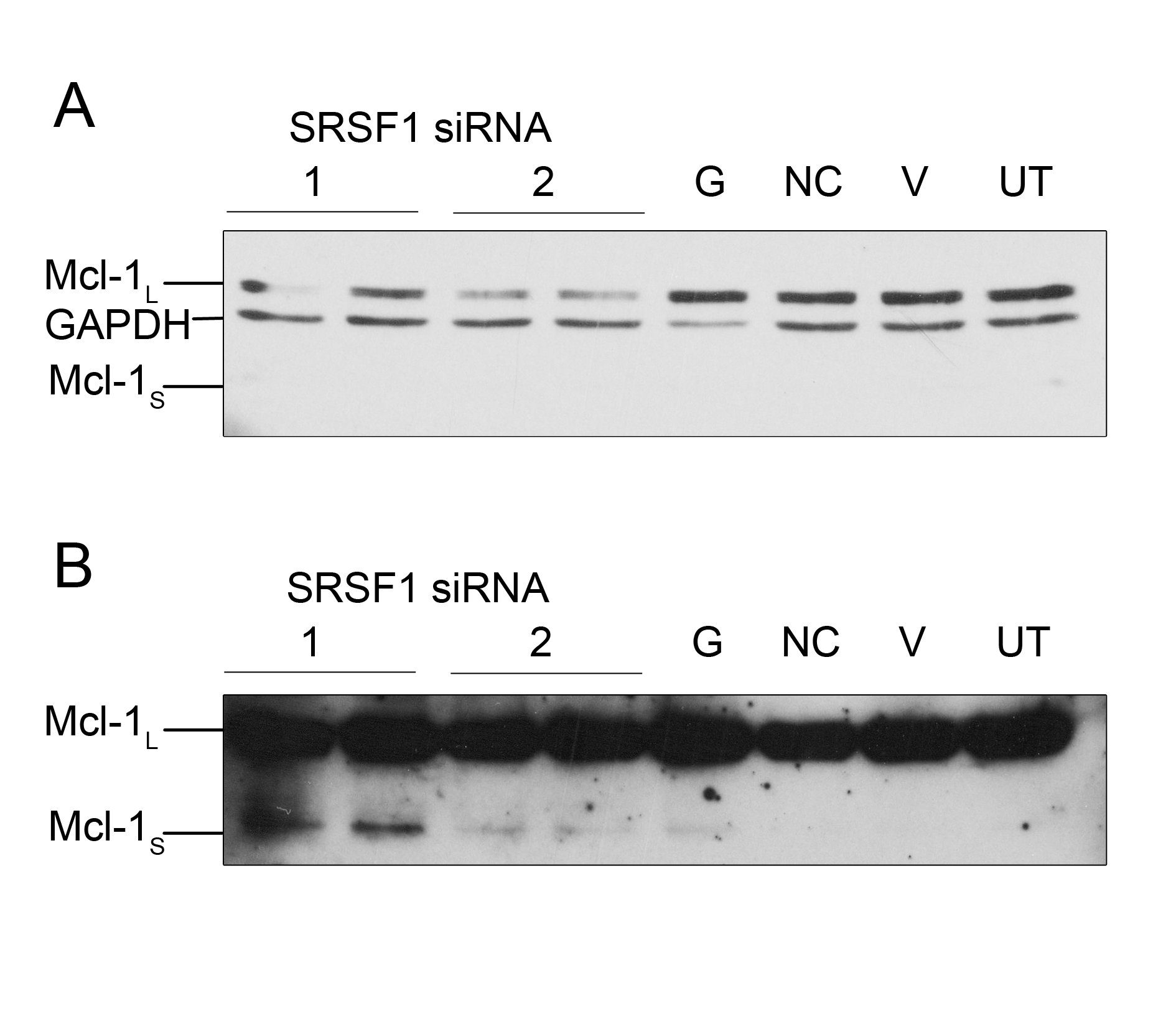

Supplement: Figure S4 — Expression of Mcl-1 proteins in MCF-7 cells. (A) Detection by immunoblotting of Mcl-1L protein and the loading control GAPDH in 40 ug of total cell lysate from MCF-7 using ECL detection reagent (Pierce). (B) Detection of Mcl-LS on the same membrane was achieved using an alternative and more sensitive procedure using SuperSignal West Femto ECL detection reagent (Pierce). (TIF) [file pone.0051497.s004.tif]
