# Supplementary material for: Regulation of Mcl-1 by SRSF1 and SRSF5 in Cancer Cells
Source: PLoS One. 2012 Dec 17;7(12):e51497. doi: 10.1371/journal.pone.0051497 (PMC3524227; doi:10.1371/journal.pone.0051497)
Supplement: Methods S1 — Immunoprecipitation and RNA Isolation. MCF-7 cells were washed in ice cold PBS and then collected in lysis buffer (10 mM Tris-HCl (pH 7.5), 150 mM NaCl, 0.5% NP-40, 1% Triton X-100) containing protease inhibitors (Sigma-Aldrich). Dynabeads Protein A (Invitrogen) were incubated for 3 hours at 4°C with 10 µg of antibody (mouse anti-SF2/ASF (SRSF1, Zymed) and mouse IgG control (Santa Cruz)) diluted in lysis buffer or with lysis buffer alone. Beads were then washed three times with lysis buffer before being incubated for 1 hour at 4°C with the cell lysate. Beads were then washed five times with lysis buffer before the immunoprecipitated RNA was collected in Trizol reagent (Invitrogen) according to manufacturer’s instructions. The RT-PCR was performed as before but half of the RNA obtained from the immunoprecipitation was used in each reaction. (DOCX) [file pone.0051497.s005.docx]

**Methods S1**

**Immunoprecipitation and RNA Isolation.**

MCF-7 cells were washed in ice cold PBS and then collected in lysis buffer (10 mM Tris-HCl (pH 7.5), 150 mM NaCl, 0.5% NP-40, 1% Triton X-100) containing protease inhibitors (Sigma-Aldrich). Dynabeads Protein A (Invitrogen) were incubated for 3 hours at 4⁰C with 10 µg of antibody (mouse anti-SF2/ASF (SRSF1, Zymed) and mouse IgG control (Santa Cruz)) diluted in lysis buffer or with lysis buffer alone. Beads were then washed three times with lysis buffer before being incubated for 1 hour at 4⁰C with the cell lysate. Beads were then washed five times with lysis buffer before the immunoprecipitated RNA was collected in Trizol reagent (Invitrogen) according to manufacturer’s instructions. The RT-PCR was performed as before but half of the RNA obtained from the immunoprecipitation was used in each reaction.
