# Supplementary material for: Regulation of Mcl-1 by SRSF1 and SRSF5 in Cancer Cells
Source: PLoS One. 2012 Dec 17;7(12):e51497. doi: 10.1371/journal.pone.0051497 (PMC3524227; doi:10.1371/journal.pone.0051497)
Supplement: Methods S2 — MTT Cell Proliferation Assay. MCF-7 cells were seeded and transfected with siRNA as before. After 72 hours proliferation was measured using TACS MTT Cell Proliferation Assay (Trevigen), according to manufacturer’s instructions. (DOCX) [file pone.0051497.s006.docx]

**Methods S2**

**MTT Cell Proliferation Assay**

MCF-7 cells were seeded and transfected with siRNA as before. After 72 hours proliferation was measured using TACS MTT Cell Proliferation Assay (Trevigen), according to manufacturer’s instructions.
